# The Role of the Urinary Bladder in the Diagnosis of Abdominal Compartment Syndrome: A Prospective Study in Trauma Patients

**DOI:** 10.7759/cureus.24238

**Published:** 2022-04-18

**Authors:** Samir Johna, Nina Bowman, Olivia Mac, Fanglong Dong, David T Wong, Michael M Neeki

**Affiliations:** 1 Surgery, Loma Linda University School of Medicine, Loma Linda, USA; 2 General Surgery, Arrowhead Regional Medical Center, Colton, USA; 3 Emergency Medicine, Arrowhead Regional Medical Center, Colton, USA; 4 Surgery, Arrowhead Regional Medical Center, Colton, USA

**Keywords:** multiorgan system failure, laparotomy, intravesical pressure, intra-abdominal pressure, abdominal compartment syndrome

## Abstract

Purpose

The aim of this study was to evaluate the accuracy of bladder pressures in the diagnosis and management of abdominal compartment syndrome (ACS).

Methods

After Institutional Review Board (IRB) approval, nine operative abdominal trauma patients were prospectively studied over an 18-month period. Bladder pressures were compared to pressures obtained from intra-operatively placed electronic transducer located in the pelvis. Statistical analysis was performed using methods described by Bland and Altman.

Results

A Bland-Altman plot was used to assess the differences between bladder and transducer pressures. There was little agreement between the two methods at low (10-15 mmHg) and high (30-70 mmHg) pressures. At higher pressures, there was a notable difference between these two types of pressure. No patients required repeated operation. One patient died from severe traumatic brain injury.

Conclusion

Measurements obtained from the urinary bladder did not agree well with those obtained from within the peritoneal cavity. Furthermore, abdominal pressures greater than 20 mmHg did not show signs of ACS in this selected population, raising doubts about the utility of using abdominal pressures alone in the management of ACS.

## Introduction

Abdominal compartment syndrome (ACS) is defined by the World Society of the Abdominal Compartment Syndrome (WSACS) as “sustained intra-abdominal pressure >20 millimeter of mercury (mmHg, with or without an abdominal perfusion pressure <60 mmHg) that is associated with new organ dysfunction/failure” [1]. It is a constellation of signs and symptoms clinically manifested by a single or multiple organ-system dysfunction, which may ultimately culminate into multiorgan system failure and death [1].

The interest of the medical community in ACS has waxed and waned since it was first described in the late 19th century [2]. As a result, no clear methodology was developed for the proper diagnosis of this syndrome until the 1980s. The intravesical pressure (IVP) was accepted as a surrogate for intra-abdominal pressure (IAP) and was adopted as the gold standard technique before it passed scientific scrutiny [3]. Based on the most recent guidelines updated in 2013, WSACS recommends the use of transbladder technique as the standard IAP measure technique [1]. However, this recommendation is “not graded,” unlike their other recommendations, based on the Grading of Recommendations, Assessment, Development, and Evaluation system [1].

To explore the agreement between the IVP and the IAP, the current study prospectively enrolled trauma patients who were triaged to Loma Linda University Medical Center and required laparotomy. The IVP was measured by the gold standard technique through the bladder to the IAP that was measured by an innovative technique.

## Materials and methods

The study protocol was reviewed and approved by the Institutional Review Board at Loma Linda University. All patients requiring laparotomy were candidates for the study except pregnant women and patients younger than 15 years of age. Patients in extremis who could not provide consent directly or through family members could not be enrolled. Over an 18-month period, nine patients were recruited for the study.

The equipment for the procedure included a three-way Foley catheter, 500-cc intravenous bag, three-way stopcock, intravenous tubing, transducer kit, and a 2-feet pressure monitoring line. The equipment was assembled by spiking the intravenous bag and priming the intravenous tubing. The intravenous tubing was then connected to one of the ports on the three-way stopcock. The transducer was connected to the two-feet pressure monitoring line and both were primed. The pressure monitoring line was connected to the remaining port on the three-way stopcock. The transducer was hooked up to the monitor and was calibrated using the symphysis pubis of the supine patient as a zero reference point. The three-way stopcock was finally connected to the irrigation port of the three-way Foley catheter.

In surgery, the three-way Foley catheter was placed after induction of anesthesia. Camino® (Integra NeuroSciences, Plainsboro, NJ, USA) transducer was zeroed in compliance with the manufacturer’s instructions prior to insertion through the left lower quadrant of each enrolled patient and was stationed in the pelvis close to the urinary bladder dome after the completion of laparotomy. Once in the ICU, the abdominal measurements were recorded every hour, whereas the urinary measurements were recorded every 4 hours. The measurements were continued for 72 hours or until the patient was transferred out of the ICU, whichever came first. The pressures were measured with the patient in the supine position and with the pressure line at the level of symphysis pubis at the end of expiration whenever possible.

All statistical analyses were conducted using the R software package “rmcorr” version 0.4.4 (R Foundation for Statistical Computing, Vienna, Austria) [4]. Repeated measures correlation was calculated for the data. A scatterplot of IAP versus IVP was produced through the plot.rmc function. A Bland-Altman analysis was also conducted to assess the agreement between IAP and IVP [5]. All statistical analyses were two-sided. A p-value of <0.05 was considered to be statistically significant.

## Results

**Figure 1:**
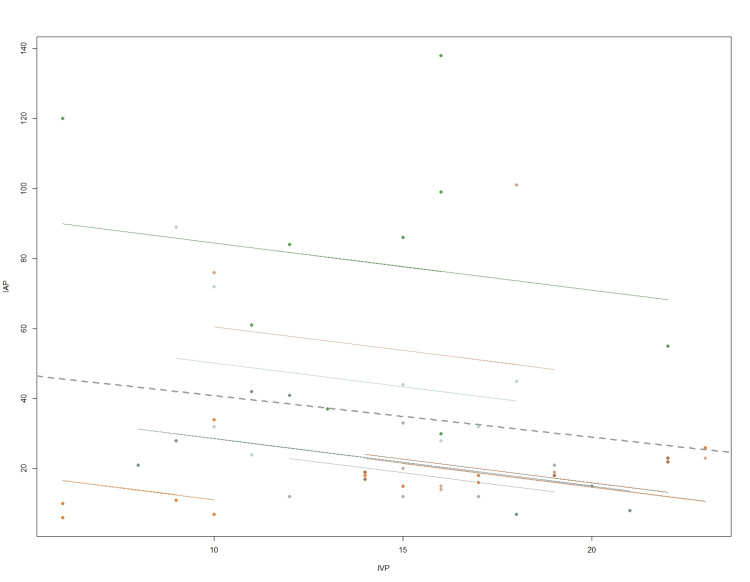
Scatterplot of IAP versus IVP IAP, intra-abdominal pressure; IVP, intravesical pressure

A total of nine patients were included in the analysis. Each patient had three to nine measures of both IAP and IVP. Figure 1 presents the scatterplot of IAP versus IVP. The repeated measures correlation coefficient was -0.2079 (95% confidence interval: -0.400 to -0.068, p = 0.004). No patient required repeated operation, and one patient died from severe traumatic brain injury.

**Figure 2:**
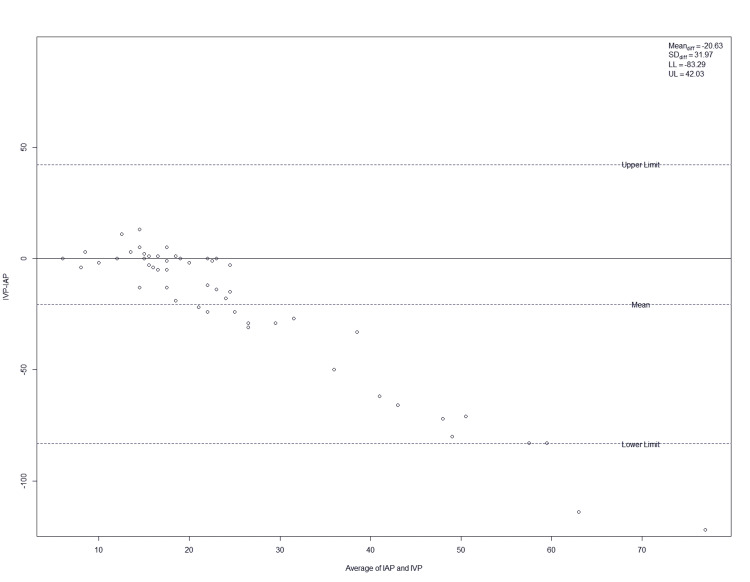
Bland-Altman plots of IAP versus IVP IAP, intra-abdominal pressure; IVP, intravesical pressure

Bland-Altman plots of all pressure differences (intravesical minus intra-abdominal) against their range of pressures obtained from nine patients are shown in Figure 2. The differences of the average pressures of each patient are shown in Figure 2. There is little agreement between the monitoring methods at low (10-15 mmHg) or high (30-70 mmHg) pressures. At higher pressures, there is a notable difference between these two types of pressure.

## Discussion

ACS is an established and well-described clinical entity that can lead to serious morbidity and mortality if the window of opportunity for timely management is missed [1]. Its diagnosis, however, is open to debate [6].

Although the measurement of IVP has been widely accepted as the gold standard technique for the diagnosis of ACS, the accuracy in predicting IAP is still a subject of controversy [6]. Hort et al. found that there was no significant correlation between IVP and need for operative intervention for ACS in neonates, but elevated IVP was associated with neonatal morbidity [7]. While many studies had shown that IVP can be used as a surrogate for IAP, a critical reevaluation of those studies uncovers some flaws in methodology as well as in statistical analysis [8,9].

In addition to the lack of standardization in materials and methods, most animal and human studies were conducted under general anesthesia, thus eliminating the role of abdominal wall musculature and its relationship to IAP [6]. As such, we cannot extrapolate the results to critical care units where patients might not be paralyzed. Likewise, studies using laparoscopy comparing IAP to IVP were conducted under general anesthesia, and, in addition, they were prone to errors by flow dynamics resulting in a rapid increase in IAP during insufflation [6].

Urodynamic studies have shown that the urinary bladder wall is not a passive diaphragm through which the abdominal pressure is transmitted into the urinary bladder. Its detrusor muscle reacts dynamically to its surroundings and generates its own pressure, which is the basis for urodynamics measurements. IVP represents a summation of the pressure caused by bladder wall events (detrusor contraction) and the pressure caused by extravesical events such as IAP. Furthermore, the bladder’s response to the filling is affected by the temperature and the pH of the injected fluid [10,11]. Likewise, rapid infusion of the fluid into the bladder may induce involuntary contractions of the detrusor muscle [12].

Previous research had accepted IVP as a surrogate for IAP after measurements from both locations were statistically analyzed using correlation coefficients. However, Bland and Altman had suggested that such analyses are misleading due to many factors [13]. To name a few, correlation coefficient measures the strength of relation between the variables but not the agreement, a change in scale of measurement does not affect the correlation but it certainly affects the agreement, and correlation depends on the range of the true quantity in the sample. Since investigators usually try to compare two methods over the whole range of the values encountered, a high correlation is almost guaranteed. Furthermore, even data that seem to be in poor agreement can produce a high correlation. Thus, Bland and Altman had rightfully proposed the use of the analysis of agreement instead [13].

Since the utility of IVP is under scrutiny, we adopted the same methodology used by prior investigators except for the abdominal compartment. Our study is different in two aspects. First, to eliminate the unpredictable effects of anesthesia and the flow dynamics of abdominal insufflation, we placed intra-abdominal Camino transducers, which enabled the collection of multiple direct and spontaneous readings over a long period of time [6]. Second, we subjected our data to the analysis of agreement instead of the correlation coefficients as proposed by Bland and Altman [13].

Our study has some limitations. Perhaps the most concerning is the small number of subjects enrolled in the study. We encountered difficulties due to the inability to obtain consent. Most trauma patients who required laparotomy were in extremis and were not accompanied by family members to be able to obtain consent. The Institutional Review Board of Loma Linda University did not grant a waiver for a window of opportunity that would have improved the chances of enrollment. This situation led to a biased selection of relatively stable patients. However, the aim of our study was not to diagnose ACS but rather to compare measurements from the two compartments.

## Conclusions

Our data showed that measurements obtained via the urinary bladder did not agree with those obtained from within the peritoneal cavity, particularly with low (10-15 mmHg) and high pressures (30-70 mmHg). The urinary bladder did not reflect elevated IAP to the same degree, nor did patients with elevated IAP > 20 mmHg show clinical signs of ACS, thus raising doubts about the utility of IVP measurement in the diagnosis of ACS. In fact, in patients with higher IAP, there was a negative bias, which indicates that the intravesical measurement underestimated the IAP. Ultimately, a solid reproducible technique for measuring IAP that is accurate and easily performed in the critical care setting is yet to be determined.
